# Maternity Care Services and Culture: A Systematic Global Mapping of Interventions

**DOI:** 10.1371/journal.pone.0108130

**Published:** 2014-09-30

**Authors:** Ernestina Coast, Eleri Jones, Anayda Portela, Samantha R. Lattof

**Affiliations:** 1 Department of Social Policy, London School of Economics and Political Science, London, United Kingdom; 2 Department of Maternal, Newborn, Child and Adolescent Health, World Health Organization, Geneva, Switzerland; UCL, United Kingdom

## Abstract

**Background:**

A vast body of global research shows that cultural factors affect the use of skilled maternity care services in diverse contexts. While interventions have sought to address this issue, the literature on these efforts has not been synthesised. This paper presents a systematic mapping of interventions that have been implemented to address cultural factors that affect women's use of skilled maternity care. It identifies and develops a map of the literature; describes the range of interventions, types of literature and study designs; and identifies knowledge gaps.

**Methods and Findings:**

Searches conducted systematically in ten electronic databases and two websites for literature published between 01/01/1990 and 28/02/2013 were combined with expert-recommended references. Potentially eligible literature included journal articles and grey literature published in English, French or Spanish. Items were screened against inclusion and exclusion criteria, yielding 96 items in the final map. Data extracted from the full text documents are presented in tables and a narrative synthesis. The results show that a diverse range of interventions has been implemented in 35 countries to address cultural factors that affect the use of skilled maternity care. Items are classified as follows: (1) service delivery models; (2) service provider interventions; (3) health education interventions; (4) participatory approaches; and (5) mental health interventions.

**Conclusions:**

The map provides a rich source of information on interventions attempted in diverse settings that might have relevance elsewhere. A range of literature was identified, from narrative descriptions of interventions to studies using randomised controlled trials to evaluate impact. Only 23 items describe studies that aim to measure intervention impact through the use of experimental or observational-analytic designs. Based on the findings, we identify avenues for further research in order to better document and measure the impact of interventions to address cultural factors that affect use of skilled maternity care.

## Introduction

Global strategies to reduce maternal and newborn mortality and health have emphasised the need for scaling up the use of skilled maternity care [1]. Yet, experience has shown that provision of skilled care and availability of maternity care facilities does not necessarily lead to increased utilisation. A large body of literature describes how cultural factors affect women's use of services [2]–[4], including those resulting from differing ‘cultures’ of maternity care between service providers and populations served [5].

### What is culture?

There is no one agreed definition of culture, but a focus on culture means emphases placed on aspects such as shared norms, beliefs and expectations, spoken language and behavioural customs [6]. In reality, it is difficult to separate out culture from social, economic and geographical context [7]. For example, members of a cultural group might not use a particular health service because they are too poor or because they know they will be discriminated against – highlighting the danger of conflating poverty with culture. Culture includes components that are both explicit and implicit. Hall [8] describes different levels of culture: a level that is explicit or manifest to outsiders (e.g., language, rituals, dress), a level of rules and norms that are known to group members but rarely shared with outsiders, and a level that is known and followed but not stated. Most societies have more than one culture within them. These cultural sub-divisions might take the form of social groups or strata (e.g., ethnic groups, religious groups, social classes, castes, ranks) marked by distinctive cultural attributes (e.g., beliefs, behaviour, perceptions, attitudes to illness and health, religion, language, manners, dress, housing, diet) alongside social and economic attributes (e.g., wealth, power, gender, education).

### Culture and maternity care services

Childbirth, and the time around birth, is a social and cultural event that is often governed by norms. However, in most societies, the dominant culture, expressed through social institutions such as the health care system, regulates how health issues are both perceived and addressed. Differences between the cultures of health care services and service users have been recognised as a major issue in service delivery. Perceived or actual cultural insensitivity or incompetence of professionals can lead to perceptions of poor quality care by users or discrimination of certain users by providers, resulting in a lack of trust in services and service providers [9]–[11].

Many authors have recommended that cultural factors should be taken into account in the planning and delivery of services in order to effectively encourage service uptake as an important step in reducing maternal and newborn mortality [5], [12]–[16]. Intercultural approaches to the design and delivery of national policies are well-established in some countries, particularly in Latin America [17]. The need for ‘culturally-appropriate’ health facilities is core to the World Health Organization's (WHO) mandate on ‘health for all’ [18] and its strategy for improving maternal and newborn health [5]. However, research reveals the complexity of such endeavours. [19]. It is known that some interventions have been implemented in different world regions to address cultural factors that affect the use of maternity care services. However, the literature has not been synthesised.

### Aims and objectives

This systematic mapping of the literature aims to understand the range of interventions that have been implemented to address cultural factors affecting women's use of skilled maternity care services. The study's objectives were to methodically identify and develop a map of the literature; categorise the range of interventions, the type of literature and the study designs included; and identify knowledge gaps.

## Methods

### Methods

The methodology for systematic mapping used in this study was developed from work at the Evidence for Policy and Practice Information and Co-ordinating Centre and is increasingly used in a range of social sciences [20]–[24]. The scope and types of literature included in a systematic mapping are normally broader than in a systematic review. The aim in this mapping is to describe as widely as possible all of the literature relating to the topic without limiting to studies that assess the strength or direction of the relationship, or even to empirical studies. We developed a protocol that was reviewed by an advisory group composed of content and method experts.

### Inclusion/exclusion criteria

Potentially eligible studies included journal articles, and published and unpublished information from governments and other agencies, whether available in print or online, published in English, French and/or Spanish. Since the aim is to describe the nature and coverage of the literature, quality was not assessed and was not a criterion for inclusion. Multiple references based on the same sample were also not excluded (as would be the case in a systematic review in order to avoid bias).

Table 1 provides the specification of the items to be mapped. Whilst intervention aims and outcomes relate only to women's use of services during pregnancy, childbirth and after birth, intervention recipients may include, for example, household or family members, community leaders or maternity care providers. Skilled care is defined in this mapping as those services provided by a skilled attendant: an “accredited health professional – such as a midwife, doctor or nurse – who has been educated and trained to proficiency in the skills needed to manage normal (uncomplicated) pregnancies, childbirth and the immediate postnatal period, and in the identification, management and referral of complications in women and newborns. Traditional birth attendants, trained or not, are excluded” [25]. Where the distinction between skilled and unskilled care is not clear, an inclusive approach is adopted. However, interventions that focused primarily on traditional birth attendants' (TBAs) roles in the direct provision of childbirth services were excluded, as the focus of the paper is on skilled maternity care services. Interventions concerned with improving satisfaction, but not also the use of services, are excluded.

**Table 1:** Specification of systematic mapping.

**Population**	The outcomes apply to women during pregnancy, childbirth and after birth in any world region. The intervention population may be broader, including other community members or health providers.
**Intervention**	An implemented intervention that is primarily and explicitly designed to accommodate or address a cultural group's shared norms, values and/or beliefs; behavioural customs; and/or spoken language/s. Cultural group is defined broadly to include any form of group or social stratum that is (considered to be) marked by its own distinctive cultural characteristics [7], [128], such as ethnic groups, groups defined by religion, language groups, indigenous groups, tribal communities, caste-based groups/strata or geographically defined populations.
**Aim/outcome**	Items must state an aim to increase, or measure as an outcome, women's use of skilled care during pregnancy, childbirth and after birth.
**Study design**	Reviews are not included. No other criteria are specified for study design.

Items must describe an *implemented* intervention in which a primary, focused aim or strategy is to address cultural factors as a vehicle to change use of maternity care services. This criterion excludes the following:

- Interventions that exclusively address economic or geographical access barriers for a defined cultural group, although overlaps between cultural factors on the one hand and economic and geographical factors on the other are acknowledged [7];
- Generic quality improvement interventions that consider and/or accommodate cultural factors explicitly or implicitly, but not as a primary, focused aim or strategy. Whilst it is acknowledged that an “important question may be what combination of interventions and ways of incorporating culture into generic quality improvement are most likely to improve quality of care and outcomes” [6], this question is beyond the scope of this mapping; and
- Items in which ‘cultural appropriateness’ is evaluated, but not incorporated in intervention design or implementation.

### Search strategy

Electronic databases, registers and websites were assessed for their availability, relevance and likely coverage of the eligible literature. Ten electronic databases and two targeted websites were included and searched for items published from 1 January 1990 to 28 February 2013. Combinations of relevant search terms were developed and tested in a sample of databases for sensitivity to a list of references that were known to the research team and judged to be potentially relevant. Table 2 presents the final combinations of search terms used. Electronic searches were adapted to each database using appropriate truncations/wildcards. Titles and abstracts were normally searched, and Medical Subject Headings (MeSH) terms were included where possible. A call for papers was sent by the WHO to various organisations and topic experts. The references received were combined with the electronic search results.

**Table 2:** Search terms and their combinations.

1. Intervention/study type terms	2. Access/use terms	3. Care terms	4. Population terms	5. Culture terms
Arrangement*	Accept*	Advice	***Antepartum terms***	Attitud*
Evaluat*	Access	ANC	Ante*natal	Behaviour*
Initiative*	Appointment*	“Birth attendan*”	Ante*partum	Behavior*
Intervention*	Attend*	Care	Expect*^a^	Belief*
Model*	Availab*	Doctor*	Pregnan*	Believ*
Package*	Obtain	Centre	Prenatal	Caste*
Pilot*	Outreach	Center	Trimester	Communit*
Program*	Recei*	Clinic*	***Intrapartum terms***	Culture*
Project*	Seek*	Counsel*	Birth*	Cultural
Provision*	Uptake	Department*	Child*birth	Custom*
Regime*	Use	Facilit*	Intra*partum	Ethnic*
Scheme*	Utilisation	Healthcare	Maternity	Indigen*
Strateg*	Utilization	Health care	Obstetric*	Language*
Trial*	Visit*	“Health system”	Parturition	Minorit*
		Hospital*	Partus	Norm*
		Institution*	Peri*natal	Race*
		Midwif*	Deliver*^a^	Racial*
		Nurs*	Labour^a^	Religio*
		Physician*	Labor^a^	Ritual*
		PNC	***Postpartum terms***	Sub*cultur*
		Service*	Maternal	Sub*population*
		Treatment*	“New mother”	Tradition*
		Unit*	Post*natal	Tribal*
			Post*partum	Tribe*
			PPC	Value*
			Puerper*	Participatory

a These terms have multiple meanings. Due to their presence in a column that narrowed a search that was otherwise very broad, they were included only in searches where their inclusion did not yield an unfeasible number of references.

Table 2 provides the basic template that was adapted to the particulars (e.g., wildcards using the asterisk symbol (*) and truncations, capacity for complex searches, MeSH facility) of each electronic database. The full protocol is available from the corresponding author or from WHO Department of Maternal, Newborn, Child and Adolescent Health at portelaa@who.int.

### Screening process

**Figure 1:**
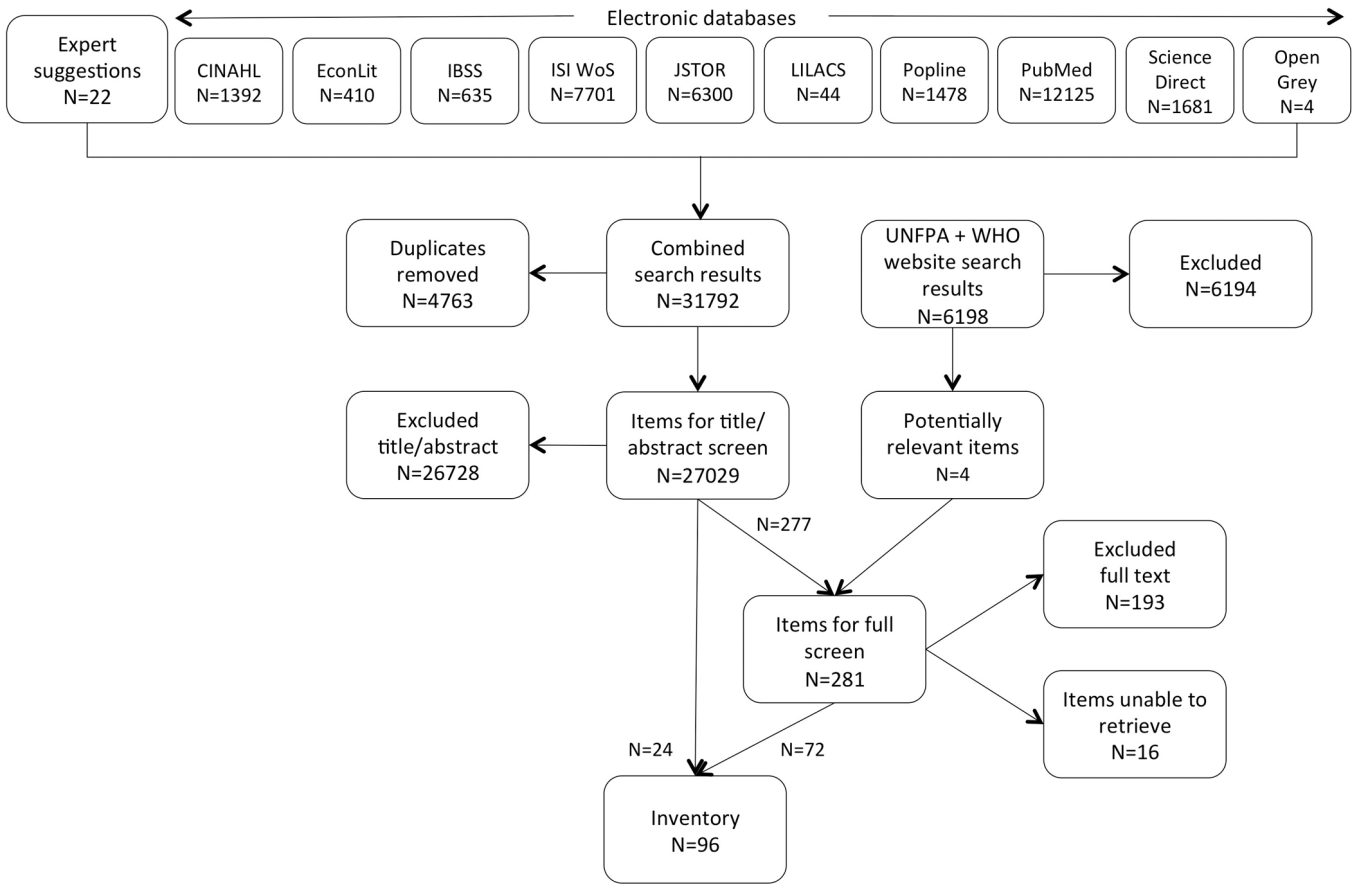
Mapping results.

All items identified through the search were screened initially on the basis of title and abstract. Where inclusion or exclusion could not be determined on the basis of title and abstract, the full text was screened. Figure 1 illustrates the screening process. EC, EJ, EH and SK contributed to screening the items individually. The following measures were taken for quality assurance: 1/all team members involved in screening independently screened the first 100 items. The whole team subsequently met to compare results, and discuss and resolve any differences in understanding of the inclusion/exclusion criteria. The criteria were further elaborated where necessary; 2/at the full text screening stage, any items that a team member considered borderline or problematic were noted. At the end of the process, EJ and EH independently screened all of the problematic items. Again, the whole team met to compare results, debate and resolve any differences. Decisions were made in favour of an inclusive approach where questions remained.

### Analysis

Data were extracted for analysis from all items in the map, including background information; a description of the intervention; details of the type of literature (and study if relevant); and details of relevant outcomes measured. Based on the map, we inductively developed and defined intervention categories. It was unfeasible to create perfectly discrete categories since many interventions are complex. Where an intervention could have fitted into more than one category, did not fit neatly into any category, or was not described in sufficient detail to understand its content, the item was placed in the category in which it was deemed to fit best. Data are presented in tables along with a narrative synthesis.

## Results

After removing duplicates, the electronic database searches and website searches, combined with references suggested by experts, generated a total of 33,227 items for screening. The majority of items were not relevant. Following the screening, a total of 96 items were included in the map. The research team was unable to retrieve or screen a further 16 items.

Interventions addressing cultural factors affecting the use of skilled maternity care are not confined to a specific type of country. The map includes items from 35 countries across all world regions and the whole range of country income levels. Thirty-nine of the items were based in high-income countries, of which the majority were from the United States of America (USA), followed by Australia, Canada and the United Kingdom (UK). Items from the USA and UK predominantly included interventions targeting specific immigrant or ethnic minority groups, whilst items from Australia and Canada predominantly targeted indigenous communities. Only one item was based in continental Europe, although this may partly reflect the systematic mapping's restricted language coverage.

Of the remaining items, 25 were from low-income countries and 29 from middle-income countries, with three items located in multiple countries that cut across these categories. Sixteen items described interventions across nine countries in Eastern and Western Africa, and 14 items described interventions in Latin America. Twenty-seven items from Asia were dominated by literature from southern Asia.

### Description of interventions

The five intervention categories we developed were: (1) service delivery models, (2) service provider interventions, (3) health education interventions, (4) participatory approaches, and (5) mental health interventions. We define the categories and describe the range of interventions within each below.

#### 1. Service delivery models

This category includes 24 items describing models of service delivery specifically designed (or adapted from existing models of service delivery) to provide culturally-appropriate services for targeted groups (Table 3). These models are largely complex interventions taking a broad range of measures, often including elements of the ‘service provider interventions’ and ‘participatory approaches’ described below as well as adaptations to the service setting, practices, materials and/or language. The category is dominated by interventions from Australia and Latin America.

**Table 3:** Included items: Service delivery models.

Author and year	Country (ies)	Intervention	Literature/study type	Maternal care outcomes measured
Affonso et al. 1993 [37]	USA (Hawaii)	Culturally-sensitive nursing care and community outreach interventions (prenatal) congruent with the lifestyles of ethnic groups in Hawaii	Narrative description of intervention	
Ahmed and Jakaria 2009 [42]	Bangladesh	Cadre of skilled birth attendants developed for home births (with a culture-based rationale).	Descriptive study (qualitative + performance monitoring surveys)	Number and proportion of women covered
Bender et al. 2005 [36]	Bolivia	Community-based intervention. Emphasis on quality care to provide culturally-acceptable services appropriate to women's needs. Used popular education in the community's meeting centre as a first point of entry.	Descriptive study (monitoring data)	Utilization of antenatal care (ANC) (number of first visits in 1st trimester; total of at least 4 visits)
Blum et al. 2006 [43]	Bangladesh	A national level initiative to post skilled birth attendants at community level (with a culture-based rationale).	Qualitative study	
Dickerson et al. 2010 [129]	China (Tibet)	A programme to train rural healthcare workers and laypersons to provide outreach to pregnant women and families (with a partly culture-based rationale), emphasizing a continuum of community-level care.	Descriptive study (survey)	Number of antepartum care visits; birth place
Eckermann and Deodato 2008 [31]	Lao PDR	Introduction of maternity waiting homes, incorporating research findings on traditional practices, and social and cultural (among other) factors	Descriptive study (formative study + monitoring data)	Number of women using facility
Gabrysch et al. 2009 [35]	Peru	Culturally-appropriate delivery care model developed in cooperation with Quechua indigenous communities and health professionals. Involves features such as a rope and bench for vertical delivery position, inclusion of family and TBAs and use of the Quechua language.	Descriptive study (formative study + survey)	Proportion of births delivered in health facility
Greenberg et al. 1996 [130]	USA	Various strategies by Washington State Department of Health to ensure access to health care for ethnic minority groups, including culturally-competent staff; targeted outreach; development of partnerships with community organizations and other agencies.	Narrative description of intervention	
Homer et al. 2012 [26]	Australia	Community outreach service developed into a comprehensive model of care known as the Malabar Community Midwifery Link Service. Prioritises Aboriginal and Torres Strait Islander communities and aims to provide culturally-appropriate services. Aboriginal Health Education Officers work alongside midwives and nurses, ensuring community engagement and cultural safety, and providing a link between the community and the service. Aboriginal midwives are mentored.	Descriptive study (clinical data + qualitative component)	Number of women accessing the service; starting ANC before 20 weeks
Jan et al. 2004 [27]	Australia	Daruk Aboriginal Medical Service is a community- controlled health service initiated by local Aboriginal communities and aiming to deliver holistic and culturally appropriate health care. Includes a full-time Aboriginal health worker and cultural awareness sessions for local hospital staff.	Observational analytic study (retrospective cohort study) + qualitative component	Gestational age at first visit; number of antenatal visits
Kenney et al. 2005 [41]	Kazakhstan	Holistic, family-centred and culturally-appropriate approach to de-medicalise care. Elements include involving women in decision-making on care; evaluating new interventions in a national context for their impact on cultural attitudes and facilitating their acceptance through information and discussion; and introduced change through understanding and agreement with local people (rather than orders).	Descriptive study (analysis of trends using clinical data, pre-post surveys and official statistics + qualitative component)	Place of ANC; provider of ANC; number of ANC visits; initiating ANC in 1st trimester; referral to specialists; prenatal hospitalisation; type of birth (normal vs complicated)
Kim 2003 [40]	Korea	Sanhujori centres to provide more systematic professionalised care for postpartum women as an alternative to traditional sanhujori postpartum care provided by family members. Centres accommodate traditional beliefs while providing quality postpartum care and support (not exclusively concerned with health).	Descriptive study (cross-sectional survey)	Number of women admitted to a care centre
Larson et al. 1992 [131]	USA	A bilingual Migrant Health Project Team assisted staff at migrant health centres in designing culturally-appropriate strategies for delivering care to migrant groups. A migrant-specific maternal care coordination programme was developed that used bilingual staff, outreach services and migrant lay health advisers.	Descriptive study (clinical data + trends in outcomes)	First trimester entry into prenatal care; receiving 9+ prenatal visits
Mayberry et al. 1999 [38]	USA (Hawaii)	The Caring for Pregnant Women health care programme (Malama Na Wahine Hapai) is purposefully built on values and customs of Native Hawaiian, Filipino, and Japanese women - ethnic groups with late or no entry into prenatal care. Complementary to standard obstetric care. Informed by focus groups, it aims to provide culturally competent care through attention to training and monitoring by local cultural and ethnic healers and neighbourhood leaders.	Narrative description of intervention	
McAree et al. 2010 [132]	UK	Midwifery group practice compared to standard maternity care in an ethnically-diverse area (with a partly culture-based rationale).	Qualitative study	
Moreno and Lopez 2013 [34]	Ecuador	National Plan adapted to Kichwa indigenous communities. It involves intercultural health strategies to overcome barriers that were mainly cultural in nature; respect for and use of indigenous knowledge and implementation of culturally-appropriate birth houses; and training of traditional Kichwa birth attendants.	Descriptive study (project output statistics)	Number of births in institutions
Nel and Pashen 2003 [28]	Australia	Service changes in local and regional ANC services. Consultation with health providers and Aboriginal communities to address cultural and family needs. The programme includes a separate indigenous medical centre managed by a community board and staffed by indigenous people. Pregnant women are seen in familiar surroundings, initially by indigenous staff. Aboriginal health workers make home visits to ensure service use. Pregnant women are allowed to bring children and family members.	Descriptive study (pre-post comparison of outcome counts)	Number of women presenting for birth without any ANC; attendance at the medical centre
Nurena 2009 [133]	Peru	Integration of an intercultural focus into health policies, in this case specifically reorienting services and including approaches to respond to and address the needs and cultural preferences of indigenous populations, focusing on respect for a vertical birth position.	Narrative description of intervention	
Panaretto et al. 2005 [29]	Australia	Collaboration with indigenous community to improve antenatal services, producing an integrated model of antenatal shared care delivered from the community-controlled Townsville Aboriginal and Islander Health Service.	Observational analytic study (Prospective cohort study)	Total visits per pregnancy; Total TAIHS ANC visits per pregnancy; weeks of gestation at first visit; pregnancies with inadequate care; pregnancies with late first visit
Panaretto et al. 2007 [30]	Australia	Sustained, community-based collaborative approach to ANC services for indigenous women.	Observational analytic study (Prospective cohort study)	ANC visits per pregnancy; gestation at first visit; pregnancies with inadequate care; pregnancies with late first visit
Reavy et al. 2012 [39]	USA	Culturally Appropriate Resources and Education (CARE) clinic is a nurse-led clinical programme providing healthcare services and education.	Descriptive study (qualitative study + clinical data)	Missed clinical appointments derived from chart reviews (all other data qualitative, although aim was to address barriers in accessing healthcare services)
Schooley et al. 2009 [32]	Guatemala	The Casa Materna maternity waiting home and community outreach strategy seeks to bridge traditional and western approaches to health care services. Built on principles of respect, caring and culturally-appropriate care, the strategy uses TBAs and volunteer health advocates, and involves men as advocates.	Qualitative study	
Tucker et al. 2013 [33]	Mexico	Intercultural birthing house established as a place where women can give birth with their TBAs with access to skilled birth attendants to reduce maternal mortality among indigenous women.	Qualitative study	
Zangari 2009 [134]	Bolivia	A strategy to culturally adapt maternal health services, through training health workers to improve intercultural care, strengthening institutional management, and developing recommendations for a model of maternal care with an intercultural focus, with local participation.	Descriptive study	

Several items from Australia describe comprehensive service delivery models for Aboriginal communities, implemented through a targeted health service [26]–[30]. For example, Nel and Pashen describe an indigenous medical centre managed by a community board and staffed by indigenous service providers [28]. Service users are seen in familiar surroundings, and Aboriginal health workers visit them to ensure attendance. Moreover, pregnant women are allowed to bring children and family members, in recognition of the cultural importance of extended family links. Several items in this category, particularly from Latin America, describe the introduction of culturally-appropriate ‘maternity waiting homes’ or ‘birthing houses’ [31]–[33]. Moreno and Lopez [34] describe this model of service delivery as part of a broad strategy to adapt a national plan in Ecuador for indigenous communities.

Different types of culturally-appropriate models of service delivery have been designed for indigenous communities [35], [36], ethnic groups [37], [38], refugees [39] and the general population [40]. For example, Gabrysch et al. [35] describe a delivery care model that was developed in Peru in cooperation with Quechua indigenous communities and health professionals, featuring a rope and bench for vertical childbirth, inclusion of family and TBAs in the service delivery process and during childbirth, and use of the Quechua language. One item in this category [41] is an outlier; in the item from Kazakhstan, the aim of a new culturally-appropriate service delivery approach is to introduce less medicalised care.

Some interventions refer to the provision of skilled home-based childbirth services explicitly in order to accommodate cultural norms [42], [43]. Several further interventions in which a culture-based rationale was cited for training TBAs were excluded since the TBA was solely responsible for the direct provision of childbirth services [44]–[49]. However, these interventions also aimed to harness the TBAs' cultural role to facilitate linkages with the formal health system and improve referral for obstetric complications.

#### 2. Service provider interventions

This category encompasses a range of interventions characterised by their focus on service providers. Analysis within this category revealed several sub-categories. One sub-category refers to three interventions in which the service provider is selected to match service users in terms of cultural characteristics [50]–[52] (Table 4). For example, Bilenko et al. [50] described an intervention in Israel in which a clinic for Bedouin families was staffed by an Arabic speaking Bedouin public health nurse. Few items describe this type of intervention as the sole focus. However, several items placed in other categories, particularly in the ‘service delivery model’ category, include this as one element of a broader intervention [26], [27].

**Table 4:** Included items: Service provider interventions.

Author and year	Country (ies)	Intervention	Literature/study type	Maternal care outcomes measured
**Service provider matched on cultural characteristics**				
Bilenko et al. 2007 [50]	Israel	A new MCH clinic in desert areas for Bedouin extended families living in tribal units, staffed by an Arabic speaking Bedouin public health nurse.	Descriptive study (review of clinical data for successive pregnancies before and after the intervention)	ANC utilisation; physician examination; at least 3 nursing visits
Dundek 2006 [51]	USA	Provision of labour support by Somali women (doulas) educated and certified in methods of labour support. The programme is based on identified barriers to culturally-competent care.	Descriptive study (clinical data and surveys)	
Nauman 1995 [52]	Mexico	The Mexican Social Security Institute (IMSS) began a rural midwife programme in a region where transport is limited and people speak indigenous languages. TBAs, IMSS-trained midwives and apprentices have a cultural role. Midwives are encouraged to use their knowledge of native herbal remedies and swap modern for traditional healthcare information.	Descriptive study (project output counts)	Number of pregnancies assisted; number of births assisted
**Bridging the gap between service providers and specific service user groups**				
Chowdhury 1998 [53]	Bangladesh	A programme to involve TBAs in a safe delivery system (with a culture-based rationale), in which the TBA becomes a catalyst for the creation of a relationship between a pregnant woman and the formal health system.	Narrative description of intervention	
Friedsam et al. 2003 [54]	USA	Addresses cultural discrimination through ‘outstationing’ Medicaid eligibility workers to community locations. Counsellors advocate on behalf of the targeted population to improve health insurance uptake.	Descriptive study (enrolment indicators)	
Hazard et al. 2009 [55]	USA	Bilingual Hispanic community women (‘Hispanic Labour Friends’) are assigned to pregnant Hispanic immigrant women to assist communication with healthcare providers and provide social support.	Qualitative study	
Hicks and Hayes 1991 [56]	UK	The Asian Mother and Baby Campaign aims to improve communication between mothers and health professionals by employing linkworkers from the same social class and background as the client.	Descriptive study (Survey of health districts)	
Julnes et al. 1994 [57]	USA	Norfolk Resource Mothers Program is a community outreach programme using ‘resource mothers’ to assist with non-medical dimensions of pregnancy and childcare, including getting prenatal care. ‘Resource mothers’ are lay people often from the culture of the adolescents.	Observational Analytic (retrospective cohort study)	Month of pregnancy when prenatal care begins; number of prenatal visits completed; place of delivery (hospital or no hospital)
Ley et al. 2011 [58]	USA	"Women of color” often forego health services perceived as intimidating and/or culturally insensitive. The Healthy Start Initiative is a major community-based programme that encourages participation. Qualified community residents are employed as outreach workers and home visitors to facilitate the delivery of culturally-competent services.	Descriptive study (survey + qualitative component)	Use of services
Marsiglia et al. 2010 [59]	USA	The Familias Sanas intervention was designed to bridge the cultural gap between Latinas and the health care system. Prenatal Partners (Compañeras) are used as cultural brokers to reinforce among pregnant Latinas the importance of the postpartum visit and of caring for one's health.	Experimental study (RCT)	Compliance with the 6–8 week postpartum visit
Mattson and Lew 1992 [60]	USA	The South East Asian Health Project (SEAHP) provides culturally-sensitive maternal and child care to south-east Asian refugees. Bilingual, bicultural outreach workers provide education about the importance of care and act as interpreters. Information is printed in three languages. Providers are educated on health care practices and beliefs of south-east Asians.	Descriptive study (survey + clinical data)	Number using the clinic; current active caseload; class attendance
Meister et al. 1992 [61]	USA	A prenatal outreach and education intervention for low-income, Hispanic women in migrant and seasonal farmworker communities. Includes a Spanish language prenatal curriculum; indigenous health promoters; and a support network of local health professionals.	Narrative description of intervention	
Parsons and Day 1992 [62]	UK	A health advocacy programme to improve obstetric outcomes in non-English speaking women. Advocates both interpret and mediate between the service users and professionals.	Observational analytic (retrospective cohort study)	Gestation at booking; non-attendance of ANC appointments; uptake of antenatal investigations
Stamp et al. 2008 [63]	Australia	Anangu Bibi Family Birthing Program provides culturally-focused perinatal care for aboriginal mothers and families, with cultural guidance from an Aboriginal Women's advocacy group. Aboriginal maternal and infant care workers take a leadership role.	Qualitative study	
Thompson et al. 1998 [64]	USA	Rural Oregon Minority Prenatal Program seeks to provide culturally-appropriate care through using a bilingual and bicultural outreach worker as a cultural broker.	Observational analytic (retrospective cohort study)	Adequacy of prenatal care utilization index, including adequacy of prenatal care initiation; adequacy of total number of visits; distribution of prenatal visits
Warrick et al. 1992 [65]	USA	Peer health workers with similar cultural and community characteristics as the target population are employed in a community-based prenatal education programme to bridge the socio-cultural gap between providers and families.	Descriptive study (survey + qualitative components)	Number of prenatal visits; trimester prenatal care began; Birth attendant
Woodard and Edouard 1992 [66]	Canada	An advisory committee is created, made up of influential native women. The programme initially employs native women as counsellors, and includes native liaison workers with language abilities and cultural knowledge.	Descriptive study (service records)	Programme coverage
**Staff training**				
Adams et al. 2005 [67]	China (Tibet)	A culturally-appropriate village birth attendant training programme. A committee ensures the curriculum used in training health workers is appropriate for the Tibetan context. Ethnographic research informed intervention design.	Qualitative study	
Andrus et al. 1997 [68]	USA	A prenatal outreach programme (Opening Doors) for a culturally-diverse population. Focusing on ‘cultural competence,’ peer health consultants work with providers to address barriers to care, and staff development and training programmes focus on cultural awareness.	Narrative description of intervention	
Bardack and Thompson 1993 [69]	USA	A prenatal programme in which medical students are taught to be humane, culturally sensitive and competent, with a focus on indigent women.	Narrative description of intervention	
Clapham et al. 2008 [70]	Nepal	An intervention to address the negative attitudes of some providers due to a combination of cultural influences and health system factors.	Descriptive study (project reviews + monitoring data)	
Fahey et al. 2013 [71]	Guatemala	PRONTO is an inter-professional obstetric and neonatal simulation training programme including components that deal with culturally-appropriate care. This intervention deals with cultural humility, informed by preliminary qualitative studies.	Formative qualitative study + narrative description of intervention	
Kreiner 2009 [72]	Canada	Initiatives to address the issue of accessible, quality maternity services to First Nation, rural and immigrant communities. Includes the development of an Aboriginal midwifery degree programme, and efforts to increase ethnic diversity and cultural competence in midwifery.	Descriptive study (qualitative + documentary sources)	
Sathar et al. 2005 [73]	Pakistan	A programme to make the individual client a focus of services and address some of the gender-related and familial constraints to health care service use.	Experimental (cluster non-RCT)	
Smith and Davies 2006 [74]	Canada	A programme to initiate the transfer and exchange of knowledge to improve prenatal care by bringing communities together to understand each other's goals and cultures.	Descriptive study (participant feedback)	

A large sub-category comprises 14 interventions in which people who share cultural characteristics with a target service user group are employed to bridge the cultural gap between this group and service providers (Table 4) [53]–[66]. They may fulfil various roles including encouraging and helping women to access care [57], [59]; assisting women in communicating with healthcare providers [55], [56]; and advocating on their behalf [62]. They are sometimes referred to as ‘linkworkers,’ ‘peer health workers’ or ‘cultural brokers’. The category is dominated by items from the USA and the UK, where interventions have been implemented with specific immigrant or ethnic minority groups. This type of intervention has a long history, with many interventions implemented as early as the 1980s and 1990s. Again, several items placed in other categories, particularly in the ‘service delivery model’ category, include this position as one element of a broader intervention [26], [32].

The final sub-category refers to eight interventions with existing staff to enhance their cultural awareness or sensitivity. These interventions have been implemented across diverse contexts and include various teaching and/or learning approaches [67]–[74] (Table 4). For example, Smith and Davies [74] describe a knowledge exchange intervention in Canada that brings "communities together to enable them to understand each other's goals and cultures."

#### 3. Health education interventions

Within this category, 22 items from diverse settings describe a wide range of strategies employed with the aim of designing culturally-appropriate health education activities (Table 5). Interventions may use one or more strategies. Several interventions use preliminary studies investigating cultural factors to inform health education activities, although they differ in the extent to which they describe how these cultural factors were addressed in the actual intervention [75]–[80]. This finding may reflect different levels of emphasis on these factors in intervention design, but may also simply be a reporting issue. For example, Opoku et al. [79] and Olaniran et al. [80] both refer to the same intervention in Nigeria. However, whilst Opoku et al. [79] make a brief mention of using research on cultural factors to inform design, Olaniran et al. [80] describe in more detail the measures taken to address cultural factors. Culturally-appropriate health education messages were developed, trainers were selected who were fluent in the local language, and campaigns were carried out in churches.

**Table 5:** Included items: Health education interventions.

Author and year	Country (ies)	Intervention	Literature/study type	Maternal care outcomes measured
Belizan et al. 1995 [76]	Argentina, Brazil, Cuba & Mexico	Prenatal care home visits where trained staff use a manual based on specially conducted ethnographic studies in each site on needs, fear, expectations and social support.	Experimental (RCT)	Utilisation of antenatal services; postpartum attendance at 40 days
Bhagat et al. 2002 [75]	Canada	Focus groups to tailor prenatal sessions to the needs and beliefs of the Punjabi community. The Punjabi language is used for materials and the event is launched during a cultural festival.	Narrative description of intervention	
Clemmons and Coulibaly 1995 [135]	Mali	The strategy used indigenous knowledge and cultural resources, including three traditional communication channels, for behaviour change (see [81]).	Descriptive study	
Clemmons and Coulibaly 1999 [81]	Mali	The strategy used indigenous knowledge and cultural resources, including three traditional communication channels, for behaviour change.	Descriptive study (survey)	Skilled birth assistance; prenatal consultations
Clemmons and Coulibaly 1999 [136]	Mali	The strategy used indigenous knowledge and cultural resources, including three traditional communication channels, for behaviour change (see [81]).	Descriptive study	
Coley 2012 [85]	USA	The Moms Matter support group employs cultural competence techniques, including the use of interpreters, hand-outs in other languages, respect for various cultures and inclusion of mothers' support people.	Qualitative study	Mother's care after childbirth
DeStephano et al. 2010 [86]	USA	A culturally-tailored health education video series for Somali women emphasising the importance of prenatal care.	Descriptive study (participant feedback + obstetric provider surveys)	
Doctor et al. 2012 [77]	Nigeria	Community education designed on the basis of a study of cultural beliefs and practices relevant to demand for antenatal care and facility-based childbirth.	Descriptive study (Baseline survey and qualitative data + preliminary evaluation with service utilisation data)	1 antenatal visit; facility-based delivery
Dynes et al. 2011 [91]	Bangladesh	A skill-based programme disseminated through a training cascade, with emphasis on respectful consideration of local knowledge and agreement on actions to be taken during an obstetric or neonatal emergency.	Descriptive study (clinical data, performance testing + qualitative component)	
Gennaro et al. 2001 [82]	Malawi	A train the trainer intervention in which village leaders teach other villagers how to improve health (with a partly culture-based rationale).	Descriptive study (cross-sectional surveys pre and post intervention)	Prenatal care; postnatal care; birth site (home, TBA, clinic, or hospital)
Gies et al. 2008 [78]	Burkina Faso	Female community leaders are trained to promote specifically-designed health messages. Messages are based on a previous socio-anthropological survey investigating local perceptions and beliefs.	Experimental study cluster (part-RCT))	3+ ANC visits; 1st ANC visit in 3rd trimester; 2+ doses IPT-SP
Gummi et al. 1997 [92]	Nigeria	Community education with key messages developed by a team of leaders and women from the community. Cultural factors to seeking care are identified and addressed, such as men's role as decision-makers.	Descriptive study (preliminary studies + clinical data + qualitative component + repeat survey)	Utilisation of obstetric services
Karl-Trummer et al. 2006 [87]	Austria, Italy	The European ‘Migrant-Friendly Hospitals’ project conducted workshops on how to design services for migrant/ethnic minority pregnant women and their families. Tailored ethno-culturally sensitive information material and prenatal training courses were developed, informed by a needs assessment.	Qualitative study	
Midhet and Becker 2010 [88]	Pakistan	An information and education programme with cultural elements incorporated in design, such as the testing of materials for suitability to the local culture and consideration of the local dynamics of decision-making power.	Experimental study (cluster-RCT)	Visiting a qualified healthcare provider during 1^st^/2^nd^ trimester for routine prenatal check-up; tetanus immunisation; type of birth attendant; in case of obstetric complication, referral to district hospital
Olaniran et al. 1997 [80]	Nigeria	Community health education and mobilisation informed by preliminary studies exploring knowledge, attitudes and practices. The intervention is discussed with community leaders and incorporates people fluent in the local dialect. Culturally-appropriate health education messages are developed in the local language or dialect, communicated through posters, drama and songs, and distributed in churches, markets and to clan heads.	Descriptive study (surveys, hospital statistics, financial records + qualitative component)	From polyclinic registers: total obstetric admissions; total deliveries; admissions for major obstetric complications
Omer et al. 2008 [89]	Pakistan	A health education intervention with participation at every stage from framing issues to planning and implementation. Communication tools are user-friendly and indigenous.	Experimental study (cluster-RCT) + qualitative component	Prenatal check-ups
Opoku et al. 1997 [79]	Ghana	Community health mobilisation, informed by preliminary studies exploring cultural and other factors. The project is discussed with the community and messages are communicated through role play and drama.	Descriptive study (health facility records, financial records, community data + qualitative component)	From hospital records: obstetric admissions
Perreira et al. 2002 [90]	Guatemala	Three health education interventions, including a clinic-based intervention using images of indigenous women with staff using buttons to identify the language they speak.	Observational analytic study (repeat cross-sectional studies)	Prenatal care utilisation
Ratnaike and Chinner 1992 [83]	Cambodia, Vietnam, Laos	A community health education system that seeks to address the unique cultural beliefs concerning medicine and health in Indochina. Cultural sensitivity and community participation are sought through minimal involvement of non-national health professionals.	Narrative description of intervention	
Sibley et al. 2001 [93]	India and Ethiopia	A community and competency-based program taking into consideration the social context of childbirth. A community self-assessment is conducted for community mobilisation and to provide information about local norms.	Narrative description of intervention	
Turan et al. 2011 [94]	Eritrea	Volunteers are trained to lead participatory educational sessions on safe motherhood.	Experimental study (non-RCT) + qualitative component + descriptive component	Birth at a health facility; % having recommended 4+ ANC visits
Yeshi et al. 2009 [84]	China (Tibet)	The programme involves village leadership in training and change promotion.	Descriptive study (project output data + survey)	Antenatal and delivery care

Related to a sub-category within ‘service provider interventions,’ one strategy within the category of health education interventions is to employ facilitators or trainers who share cultural characteristics with the relevant population [80]–[84]. For example, both Gennaro et al. [82] and Yeshi et al. [84] describe interventions employing village leaders as trainers.

Several items describe developing culturally-appropriate health education materials and practices, in terms of messages, language, modalities and/or images [80], [85]–[90]. For example, Omer et al. [89] describe using an education tool in Pakistan that reflects local materials and skills in embroidery, and DeStephano et al. [86] describe a culturally-tailored health education video series for Somali women in the USA. Finally, health education interventions have used participatory approaches [75], [84], [91]–[94]. These are distinguished from the ‘participatory approaches’ category on the basis that their primary focus is the delivery of education interventions.

#### 4. Participatory approaches

An inclusive approach was adopted for participatory interventions in this systematic mapping because a population's participation in intervention design and implementation may be considered inherently to address or accommodate cultural factors. Thus, all items in which the population's participation in intervention design and implementation was a primary and explicit strategy were included. Analysis of items in this category indicates different levels of explicit emphasis on ‘cultural’ factors, a distinction that is highlighted with sub-categories (Table 6).

**Table 6:** Included items: Participatory approaches.

Author and year	Country (ies)	Intervention	Literature/study type	Maternal care outcomes measured
**Approaches that explicitly focus on cultural factors**				
Bhattacharyya and Murray 2000 [95]	Ethiopia	A community assessment for health staff and communities to jointly identify and prioritise health problems and develop plans to solve them.	Descriptive study (pre/post intervention surveys + qualitative component)	At least 2 ANC visits for last pregnancy
Hounton et al. 2009 [96]	Burkina Faso	Community mobilisation strategies include building awareness through engaging local traditional healers, administrative and religious leaders. Capacity is strengthened through facilitating community problem-solving, and culturally sensitive and locally acceptable approaches are identified to address barriers to care.	Experimental study (cluster non-RCT)	Institutional births
Jewell and Russell 2000 [97]	USA	Minority communities form a state-wide network of grassroots county minority health coalitions and a state coalition. Coalitions develop projects to increase access to early prenatal care for minority women with the intent of eliminating cultural barriers to care. Strategies include use of minority professional and paraprofessional staff, and monitoring of projects by minority health coalition boards.	Observational Analytic study (retrospective cohort study)	Kessner Adequacy of Prenatal Care Index (including timing of 1^st^ prenatal visit; number of prenatal visits); Adequacy of Prenatal Care Utilization (including month prenatal care begins; ratio of actual number of visits to expected number)
Morrison et al. 2008 [98]	Nepal	A participatory, community-based women's group intervention informed by formative research. Aiming for sensitivity to local needs and beliefs, it addresses negotiation within families, and tailors information to the needs of local groups and stakeholders such as mothers-in-law and traditional healers. It aims to be a forum in which to discuss cultural ideas.	Descriptive study (survey, health service audit + qualitative component)	
**Approaches that refer to cultural factors affecting use only**				
Orozco-Nuñez et al. [102]		A key action was the coordination of a network to strengthen the links between communities,local authorities and health services.	Qualitative study	
Kaur 1994 [99]	Malaysia	The intervention is informed by an in-depth survey of the community's culture, beliefs and health status. Participatory planning meetings are held with relevant agencies.	Narrative description of intervention	
Mushi et al. 2010 [100]	Tanzania	In collaboration with villagers, representatives and health service providers, a community-based intervention package for Safe Motherhood is developed, relying heavily on community volunteers.	Descriptive study (surveillance data + qualitative component)	Deliveries with a skilled birth attendant; ANC attendance
O′Rourke et al. 1998 [101]	Bolivia	The Warmi project involves participatory women's groups, which identify problems and implement an action plan to address them.	Observational analytic study (case control study pre/post intervention)	Received prenatal care; utilisation of trained birth attendants (including TBAs, health promoters, physicians and nurses)
**Approaches that inherently address cultural factors**				
Afsana 2012 [103]	Bangladesh	BRAC develops partnerships with communities to develop capacity and to build human resources for health at the community level.	Descriptive study (district indicators)	Receiving 4+ antenatal visits; referral to hospital for problems; place of delivery; 3+ postnatal visits
Ahluwalia et al. 2003 [104]	Tanzania	The Community Capacity Building and Empowerment Project is designed to promote problem solving in part through increasing participation by community members in planning and decision making.	Descriptive study (formative research + monitoring data, survey)	Timely referral to appropriate health facilities
Azad et al. 2010 [105]	Bangladesh	Participatory women's learning and action cycle groups.	Experimental study (cluster-RCT)	Uptake of antenatal and delivery services; health-care seeking behaviour (for any maternal illness or complication)
Kaseje et al. 2010 [106]	Kenya	A model engaging both the service delivery system and communities in an iterative process of dialogue for assessment, planning and action.	Experimental study (cluster non-RCT) + qualitative component + descriptive component	4+ ANC attendance; health facility childbirth
Keyser and Pincus 2010 [107]	USA	A learning collaborative made up of various stakeholders, families and researchers is convened with the goal of building a model system of care.	Descriptive study (performance measures + qualitative component)	Programme enrolment; screening; referral; completed referral
Kwast 1995 [108]	Bolivia, Indonesia, Uganda, Nigeria, Guatemala	The MotherCare Project strengthened maternal health and family planning programmes through policy reform, behaviour change, and improved service delivery.	Descriptive study (case report)	ANC; trained birth attendant use
Lewycka et al. 2010 [109]	Malawi	Community mobilisation through women's groups.	Experimental study (cluster-RCT)	ANC (use of malaria prophylaxis in pregnancy + tetanus toxoid); facility based skilled attendant; uptake of prevention of mother-to-child transmission; postpartum check-ups; referral patterns.
Manandhar et al. 2004 [110]	Nepal	Participatory intervention with women's groups.	Experimental (cluster-RCT)	Any ANC; visited health facility in event of illness; institutional delivery
More et al. 2012 [111]	India	Participatory intervention with women's groups.	Experimental (cluster-RCT)	1st antenatal visit before 3rd trimester; 3 or more ANC visits; institutional delivery; postnatal check
Morrison et al. 2011 [112]	Nepal	Community mobilisation through women's groups.	Protocol for cluster-RCT	None presented (but will measure Primary- 1. Deliveries conducted by trained health workers. 2. Institutional deliveries; Secondary- 1. ANC uptake. 2. Postnatal care uptake.)
Osrin et al. 2003 [116]	Nepal	A community-level participatory intervention with mothers and other key members of the community.	Reporting on implementation, but not results, of a cluster-RCT	NA (evaluation results not reported. Will include ANC service use, health-care seeking behaviour)
Rath et al. 2010 [113]	India	Participatory intervention with women's groups.	Descriptive study (qualitative component + monitoring data)	No
Skinner and Rathavy 2009 [114]	Cambodia	A community-development approach to birth preparedness.	Descriptive study (qualitative component + provider statistics + financial data)	Number receiving ANC, Number who gave birth with health centre midwife, Number who gave birth with a TBA, Number of referrals to hospital
Tripathy et al. 2010 [115]	India	A participatory intervention with women's groups.	Experimental study (cluster-RCT)	Any ANC; 3+ ANC visits; visited health facility in case of illness during pregnancy; institutional; birth attended by formal provider

The first sub-category comprises four items that describe the participatory approach adopted as an explicit strategy to address or accommodate cultural factors affecting the use of skilled maternity care [95]–[98]. For example, Hounton et al. [96] describe their intervention in Burkina Faso as one that involved “investment in communities through an understanding of their social structure and health seeking behaviours, through identification and partnership with credible community leaders, and through identification of culturally-sensitive and locally-acceptable approaches to address transport and referrals.” Jewell and Russell [97] describe another approach implemented in the USA of forming a state-wide network of grassroots county minority health coalitions to develop projects to eliminate cultural barriers to prenatal care for minority women.

The second sub-category includes four items [99]–[102] that explicitly refer to cultural factors affecting use of skilled maternity care but do not explicitly describe whether or how the intervention addressed these cultural factors.

While the final sub-category comprises 14 items [103]–[116] that neither discuss cultural factors affecting the use of care nor explicitly frame the intervention as a strategy to address cultural factors, they are mentioned because participatory interventions are considered within the inclusive approach of this mapping as ones that inherently accommodate cultural factors.

Manandhar et al. [110] and Osrin et al. [116] describe a participatory, community-based women's group intervention with a marginalised population with low access to services in Nepal, facilitated through a community action cycle. They do not frame the intervention as one designed to address cultural factors. Yet, Morrison et al. [98], referring to the same intervention in Nepal, was placed in the subcategory of ‘participatory approaches that focus explicitly on cultural factors,’ because the item describes formative research that was used to design the intervention to be acceptable and sensitive to the local culture. The implication is that the distinction between the three sub-categories may not always be a reflection of differences in intervention content; it may in some cases simply be a result of the emphasis in authors' reporting. This attests to the elusiveness of how culture is incorporated and addressed in many health interventions.

#### 5. Mental health interventions

This small category includes three fairly recent interventions focusing on perinatal depression, all aiming to overcome treatment barriers for Latina women in the USA [117]–[119] (Table 7). All three items describe adaptations to existing interventions and models. They use strategies for addressing cultural factors that are similar to those used in other categories, such as service providers sharing cultural characteristics of the target group, a more appropriate service setting, and/or more appropriate materials or therapy in terms of language or content.

**Table 7:** Included items: Mental health interventions.

Author and year	Country (ies)	Intervention	Literature/study type	Maternal care outcomes measured
Baker-Ericzen et al. 2012 [117]	USA	A culturally-sensitive, short-term telemedicine, collaborative care intervention for addressing maternal depression among Mexican-American mothers during the perinatal period. Elements include bilingual, bicultural Mexican-American staff; training on Latina mental health issues; practices adapted to the Latino community; and intervention modules on sociocultural influences.	Descriptive study (qualitative component + monitoring data)	Treatment engagement; treatment adherence
Grote et al. 2004 [118]	USA	A culturally-relevant version of brief interpersonal psychotherapy (IPT-B) for perinatal depression. Additions include a pre-treatment engagement strategy consisting of an ethnographic interview accompanied by psycho-education to engage women in treatment; convenient service delivery location; flexible scheduling of treatment sessions at the clinic or on the phone; and facilitation of access to social services.	Qualitative study	
Le et al. 2010 [119]	USA	Cultural adaptation of an evidence-based cognitive–behavioural therapy intervention to prevent perinatal depression. Culturally-specific topics are addressed, such as immigration status, acculturation, and discrimination, and the discussion is tailored to Latinas' personal realities. Manuals are developed in Spanish and English.	Narrative description of intervention	

### Description of types of literature and study designs

The map includes a wide range of literature, from narrative descriptions of interventions to studies using randomised controlled trials (RCTs) to evaluate impact. Eleven items provide only narrative descriptions of the intervention, including details of the design process, the content or its implementation. Some items report on studies used to inform the design of an intervention now being implemented.

All other items present some type of evaluation data, whether for monitoring, outcome evaluation or impact evaluation. The majority of items present various forms of quantitative or qualitative data used for monitoring the intervention and/or to evaluate outcomes. Only 23 items describe studies that aim to measure intervention impact through the use of experimental or observational-analytic designs. Of these, 14 use experimental designs including nine RCTs and five non-RCTs, and nine use observational-analytic designs including seven cohort studies.

Differences in study designs are evident across intervention categories. A large proportion of the studies with designs that aim to measure impact, particularly those with experimental designs, are in the ‘participatory approaches’ category. This is followed by a smaller proportion in the ‘health education interventions’ and ‘service provider interventions’ categories, and very few in the other categories combined.

## Discussion

Global recognition of the need to address cultural factors affecting the use of skilled maternity care has led to a wide range of interventions being implemented across diverse settings in all world regions and across all country income levels. Overall, the map provides a rich source of information on the types of intervention options that have been attempted to address or accommodate cultural factors that affect the use of skilled maternity care. The articles included in the systematic mapping and others indicate a growing awareness of the need to incorporate culture into the design of appropriate care to improve maternal and newborn outcomes [120], [121] and to deliver more responsive, effective maternity care services.

The mapping reveals examples of good practice and success stories. However, some of the literature identified, whilst stating a clear aim of addressing cultural factors, provides insufficient detail to understand exactly how they were addressed. It is clear that the complexities of formulating and implementing culturally-responsive programmes remain [16], [122]–[126]. In some of the literature found, culture is positioned as a barrier to maternal health service use, rather than an attribute of the population that services seek to serve. The type of cultural groups in a setting and the nature of cultural factors that affect use of skilled maternity care are context-specific, and give rise to a need for different kinds of intervention approaches that both anticipate and respect a community's culture, values and beliefs. This finding is reflected in the map by the clustering of items from specific contexts in specific categories of intervention.

Cultural beliefs and behaviour are impossible to isolate from the social and economic context in which they occur [7]. Interventions research around culture and maternal health service use is heavily weighted in favour of evidence focusing on sub-populations in high-income countries. We know that 99% of maternal deaths occur in low income settings and that scaling up the use of skilled maternity care will reduce global maternal morbidity and mortality [18]. We know that focusing only on the supply side of maternity care does not necessarily lead to increased use, and that culture is often invoked as an explanation for this supply-demand gap. Our systematic mapping shows that there is a critical need for better documentation of interventions, with an emphasis on lower-income countries, and better study methods to evaluate the ways in which cultural factors can be systematically mainstreamed into programmes to increase maternity care service use. Literature on interventions addressing cultural factors as evaluated models of practice remains limited. This finding may be to some extent related to the small-scale, context-specific nature of many interventions of this type.

We exclude several interventions with TBAs providing childbirth services because our focus is on use of skilled maternal health care (see category 1). However, within these items, there are several examples where the TBAs cultural role is also harnessed to facilitate linkages with formal health services. Although WHO guidance has moved away from promoting approaches that involve TBAs in the direct provision of care at birth, it also emphasises the importance of building links with TBAs and finding new roles so that this valuable resource can continue to support women during pregnancy, childbirth and after birth, and serve as an important link between services and communities [127]. Future interventions incorporating WHO guidance on the need for women to be attended by health care workers with the appropriate skills and training may consider employing traditional health workers in alternative roles. Going beyond public health programmes, there is also a need to incorporate cultural knowledge and responsiveness into health education, the development of health policies, and the delivery of culturally-competent health care.

### Limitations

Limitations arising from the inevitable conceptual challenges of any attempt to map or categorise items focusing on ‘culture’ are acknowledged. Culture is a complex, elusive concept that is challenging to define, leading to diverse definitions and operationalisations in the literature. Usages of the term are not always helpful, and the concept is susceptible to assumptions and over-generalisation. Making distinctions between interventions that address cultural factors and those that address other factors that affect use of skilled care was not a straightforward task. Distinguishing between interventions that address cultural factors as the primary and explicit aim on the one hand, and those in which cultural sensitivity is incorporated as part of generic quality improvement on the other, inevitably involves subjective judgement.

One particular challenge was the research question's focus not on what was actually ‘done’ in the intervention, but rather on what the intervention sought to address, which was reflected in the search terms. However, what the intervention seeks to address is not always carefully described by authors.

The above conceptual challenges all had implications for what was eventually identified and included. Identifying relevant items depended in part on the research team's judgement of where the line should be drawn around those to be included (outlined in Table 1). This effort was the first attempt of its kind to map such interventions; however, the range of interventions was unknown at the design stage, making it necessary for the research team to confer frequently and to continue drawing lines throughout the screening process. Moreover, at both the searching and screening stages, identifying relevant items depended in part on how authors of potentially relevant items framed their reporting, and also on the research team's ability to overcome challenges to identifying (from the text available) those that meet the criteria. The research team was aware of the challenges at the outset and continuously sought to address them from the design stage through to the final synthesis. Nevertheless, any shortcomings remain a limitation of this systematic mapping.

Relevant items may have been missed through method limitations. More specifically, any relevant literature in languages other than English, French or Spanish would have been missed. Because searches were not designed to focus on participatory approaches, some literature on these interventions may have been missed. Also, since it would have been unfeasible to search all of the infinite potential sources of grey literature, the possibility that some items of this type were missed is high. The possibility that some in-service reports on small-scale interventions that were not intended or prepared for wider circulation were missed is particularly high. Finally, we were unable to retrieve 16 items for screening that may have been eligible for inclusion. These 16 items largely represented interventions implemented in low- and middle-income countries.

## Conclusion

The map provides a rich source of information on interventions attempted in diverse settings that might have relevance elsewhere. However, many sources lack sufficient description or robust designs that allow us to draw firm conclusions. This may be to some extent related to the small-scale, context-specific nature of interventions of this type. Addressing the impacts of interventions to address cultural factors affecting the use of maternity care services is an issue of importance for researchers, programmers, and policy makers. It requires an inter-disciplinary approach and active dialogue with communities in order to understand their cultural systems, health beliefs, health practices and preferences. In order to better serve the varied needs of communities with culturally-diverse populations, the following recommendations are made for future research and reviews:

- The interventions in this map are inherently context-specific. Nevertheless, further intervention studies with harmonised outcomes, appropriate research methods and robust designs are warranted, which may provide valuable evidence on the impact (including benefits or potential harms) of a type of intervention model.
- Where an intervention is designed explicitly to address cultural factors, sufficient detail should be provided in reporting for the audience to understand how they were addressed (i.e., by specifying the links between the cultural factors identified and the content of the intervention).
- A full systematic review may be warranted of the more cohesive set of interventions designed to provide culturally-appropriate skilled maternity care for defined ethno-linguistic or religious groups. This would allow both the quality and outcomes of intervention studies to be examined.
